# A Historical Overview of Natural Products in Drug Discovery

**DOI:** 10.3390/metabo2020303

**Published:** 2012-04-16

**Authors:** Daniel A. Dias, Sylvia Urban, Ute Roessner

**Affiliations:** 1 Metabolomics Australia, School of Botany, The University of Melbourne, Parkville, Victoria 3010, Australia; 2 School of Applied Sciences (Discipline of Applied Chemistry), Health Innovations Research Institute (HIRi) RMIT University, G.P.O. Box 2476V, Melbourne, Victoria 3001, Australia; 3 Australian Centre for Plant Functional Genomics, School of Botany, The University of Melbourne, Parkville, 3010, Victoria, Australia

**Keywords:** natural products, secondary metabolites, drug discovery, bioactivity, metabolomics, dereplication, plants, sponges, algae, fungi

## Abstract

Historically, natural products have been used since ancient times and in folklore for the treatment of many diseases and illnesses. Classical natural product chemistry methodologies enabled a vast array of bioactive secondary metabolites from terrestrial and marine sources to be discovered. Many of these natural products have gone on to become current drug candidates. This brief review aims to highlight historically significant bioactive marine and terrestrial natural products, their use in folklore and dereplication techniques to rapidly facilitate their discovery. Furthermore a discussion of how natural product chemistry has resulted in the identification of many drug candidates; the application of advanced hyphenated spectroscopic techniques to aid in their discovery, the future of natural product chemistry and finally adopting metabolomic profiling and dereplication approaches for the comprehensive study of natural product extracts will be discussed.

## 1. Introduction

### 1.1. Natural Products in History

Natural products (secondary metabolites) have been the most successful source of potential drug leads [1,2,3,4,5]. However, their recent implementation in drug discovery and development efforts have somewhat demonstrated a decline in interest [1]. Nevertheless, natural products continue to provide unique structural diversity in comparison to standard combinatorial chemistry, which presents opportunities for discovering mainly novel low molecular weight lead compounds. Since less than 10% of the world’s biodiversity has been evaluated for potential biological activity, many more useful natural lead compounds await discovery with the challenge being how to access this natural chemical diversity [3].

The earliest records of natural products were depicted on clay tablets in cuneiform from Mesopotamia (2600 B.C.) which documented oils from *Cupressus sempervirens* (Cypress) and *Commiphora* species (myrrh) which are still used today to treat coughs, colds and inflammation [3]. The Ebers Papyrus (2900 B.C.) is an Egyptian pharmaceutical record, which documents over 700 plant-based drugs ranging from gargles, pills, infusions, to ointments. The Chinese Materia Medica (1100 B.C.) (Wu Shi Er Bing Fang, contains 52 prescriptions), Shennong Herbal (~100 B.C., 365 drugs) and the Tang Herbal (659 A.D., 850 drugs) are documented records of the uses of natural products [3]. The Greek physician, Dioscorides, (100 A.D.), recorded the collection, storage and the uses of medicinal herbs, whilst the Greek philosopher and natural scientist, Theophrastus (~300 B.C.) dealt with medicinal herbs. During the Dark and Middle Ages the monasteries in England, Ireland, France and Germany preserved this Western knowledge whilst the Arabs preserved the Greco-Roman knowledge and expanded the uses of their own resources, together with Chinese and Indian herbs unfamiliar to the Greco-Roman world [3]. It was the Arabs who were the first to privately own pharmacies (8th century) with Avicenna, a Persian pharmacist, physician, philosopher and poet, contributing much to the sciences of pharmacy and medicine through works such as the *Canon Medicinae* [3].

### 1.2. Medicinal Plants in Folklore

The use of natural products as medicines has been described throughout history in the form of traditional medicines, remedies, potions and oils with many of these bioactive natural products still being unidentified. The dominant source of knowledge of natural product uses from medicinal plants is a result of man experimenting by trial and error for hundreds of centuries through palatability trials or untimely deaths, searching for available foods for the treatment of diseases [6,7]. One example involves the plant genus *Salvia* which grows throughout the southwestern region of the United States as well as northwestern Mexico and which was used by Indian tribes of southern California as an aid in childbirth [6]. Male newborn babies were “cooked” in the hot *Salvia* ashes as it was believed that these babies consistently grew to be the strongest and healthiest members of their respective tribes and are claimed to have been immune from all respiratory ailments for life [6].

The plant, *Alhagi maurorum* Medik (Camels thorn) secretes a sweet, gummy material from the stems and leaves during hot days [8]. This gummy sap is called “*manna*” and consists mostly of melezitose, sucrose and invert sugar and it has been documented and claimed by the Ayurvedic people that the plant aids in the treatment of anorexia, constipation, dermatosis, epistaxis, fever, leprosy, and obesity [8]. It was also used by the Israelis who boiled the roots and drank the extract as it stopped bloody diarrhea. The Konkani people smoked the plant for the treatment of asthma, whilst the Romans used the plant for nasal polyps [8]. The plant *Ligusticum scoticum* Linnaeus found in Northern Europe and Eastern North America was eaten raw first thing in the morning and was believed to protect a person from daily infection [9]; the root was a cure for flatulence [10,11,12], an aphrodisiac [12] and was used as a sedative in the Faeroe Islands [10,13]. *Atropa belladonna* Linnaeus (deadly nightshade) is found in central and Southern Europe, Western Asia, North Africa, North America and New Zealand. Its notoriously poisonous nature (three berries are sufficient to kill a child) firmly excluded it from the folk medicine compilation and seemed to have been accepted as dangerous to handle or to experiment with [14].

### 1.3. Medicinal Natural Products from Other Sources Used in Folklore

The fungus *Piptoporus betulinus*, which grows on birches was steamed to produce charcoal, valued as an antiseptic and disinfectant [15]. Strips of *P. betulinus* were cut and used for staunching bleeding and were also found to make very comfortable corn pads [16]. Another example is the fungus *Agaricus campestris* Linnaeux ex Fries (field mushroom) found in the northern and southern temperate zones and the Caribbean. *A. campestris*, had reportedly been stewed in milk to soothe cancer of the throat [17].

As early as the 17th–18th century, lichens had been used as dyes and were far more valued than oriental spices. To date there are no lichen derived drugs approved on the market but their applications in folklore has been well documented [18]. Lichens have been used as the raw materials for perfumes and cosmetics, medicine from the time of the early Chinese and Egyptian civilizations [19]. Well known examples include *Usnea dillenius* ex Adanson which was traditionally used for curing diseases of the scalp and is still sold in pharmacies as an ingredient in anti-dandruff shampoos and in Ireland to treat sore eyes [19]. The lichen *U. subfloridana* Stirton was mixed with tobacco and butter, boiled and then cooled and applied as a lotion [14]. *Parmelia omphalodes* (Linnaeus) Acharius, which is abundant in the British Isles, was used in brown dyes. In the highlands it was traditionally sprinkled on stockings at the start of a journey to prevent inflammation of the feet [20,21] and in Ireland it was used as a cure for bad sores under the chin as well as for burns and cuts [14].

By comparison, the marine environment has very few reported applications in traditional medicine. The red algae *Chondrus crispus* and *Mastocarpus stellatus* were sources of a beverage, which was popular as a folk cure for colds, sore throats, chest infections including tuberculosis. The alga was also boiled in milk or water and used for kidney trouble and burns [22,23]. Furthermore, three spoonfuls of the juice of the red alga *Porphyra umbilicalis* (Linnaeus) Kützing, taken every morning followed by fasting for three weeks was found to be effective against cancers, in particular breast cancer [24]. *P. umbilicalis* has also been described in the Aran Islands for easing indigestion. and was also boiled and given to cows to relieve their springtime constipation [10,25].

### 1.4. Primary and Secondary Metabolites (Natural Products)

The biosynthesis and breakdown of proteins, fats, nucleic acids and carbohydrates, which are essential to all living organisms, is known as primary metabolism with the compounds involved in the pathways known as “*primary metabolites”* [26]. The mechanism by which an organism biosynthesizes compounds called *‛secondary metabolites’* (natural products) is often found to be unique to an organism or is an expression of the individuality of a species and is referred to as “*secondary metabolism*” [26,27]. Secondary metabolites are generally not essential for the growth, development or reproduction of an organism and are produced either as a result of the organism adapting to its surrounding environment or are produced to act as a possible defense mechanism against predators to assist in the survival of the organism [26,28]. The biosynthesis of secondary metabolites is derived from the fundamental processes of photosynthesis, glycolysis and the Krebs cycle to afford biosynthetic intermediates which, ultimately, results in the formation of secondary metabolites also known as natural products [26]. It can be seen that although the number of building blocks are limited, the formation of novel secondary metabolites is infinite. The most important building blocks employed in the biosynthesis of secondary metabolites are those derived from the intermediates: Acetyl coenzyme A (acetyl-CoA), shikimic acid, mevalonic acid and 1-deoxyxylulose-5-phosphate. They are involved in countless biosynthetic pathways, involving numerous different mechanisms and reactions (e.g., alkylation, decarboxylation, aldol, Claisen and Schiff base formation [26].

It is hypothesized that secondary metabolism utilizes amino acids and the acetate and shikimate pathways to produce “*shunt metabolites*” (intermediates) that have adopted an alternate biosynthetic route, leading to the biosynthesis of secondary metabolites [29]. Modifications in the biosynthetic pathways may be due to natural causes (e.g., viruses or environmental changes) or unnatural causes (e.g., chemical or radiation) in an effort to adapt or provide longevity for the organism [29]. It is the unique biosynthesis of these natural products, produced by the countless number of terrestrial and marine organisms, which provides the characteristic chemical structures that possess an array of biological activities.

## 2. Historically Important Natural Products

**Figure 1:**
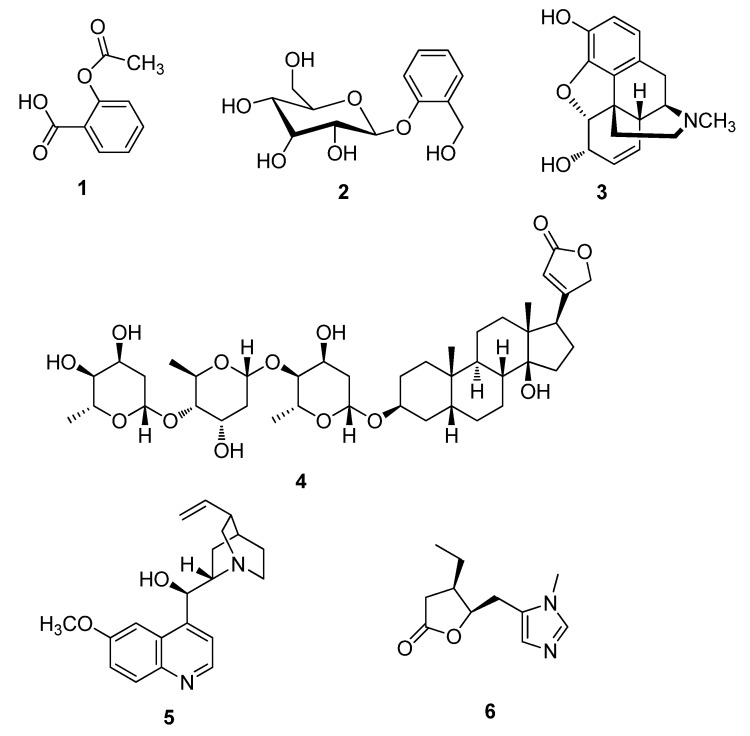
Acetylsalicyclic acid (**1**), Salicin (**2**), Morphine (**3**), Digitoxin (**4**), Quinine (**5**) and Pilocarpine (**6**).

Traditional medicinal practices have formed the basis of most of the early medicines followed by subsequent clinical, pharmacological and chemical studies [5]. Probably the most famous and well known example to date would be the synthesis of the anti-inflammatory agent, acetylsalicyclic acid (**1**) (aspirin) derived from the natural product, salicin (**2**) isolated from the bark of the willow tree *Salix alba* L. [30]. Investigation of *Papaver somniferum* L. (opium poppy) resulted in the isolation of several alkaloids including morphine (**3**), a commercially important drug, first reported in 1803 (Figure 1). It was in the 1870s that crude morphine derived from the plant *P. somniferum*, was boiled in acetic anhydride to yield diacetylmorphine (heroin) and found to be readily converted to codeine (painkiller). Historically, it is documented that the Sumerians and Ancient Greeks used poppy extracts medicinally, whilst the Arabs described opium to be addictive [30]. *Digitalis purpurea* L. (foxglove) had been traced back to Europe in the 10th century but it was not until the 1700s that the active constituent digitoxin (**4**), a cardiotonic glycoside was found to enhance cardiac conduction, thereby improving the strength of cardiac contractibility. Digitoxin (**4**) and its analogues have long been used in the management of congestive heart failure and have possible long term detrimental effects and are being replaced by other medicines in the treatment of “heart deficiency” [30]. The anti-malarial drug quinine (**5**) approved by the United States FDA in 2004, isolated from the bark of *Cinchona succirubra* Pav. ex Klotsch, had been used for centuries for the treatment of malaria, fever, indigestion, mouth and throat diseases and cancer. Formal use of the bark to treat malaria was established in the mid 1800s when the British began the worldwide cultivation of the plant [30]. Pilocarpine (**6**) found in *Pilocarpus jaborandi* (Rutaceae) is an L-histidine-derived alkaloid, which has been used as a clinical drug in the treatment of chronic open-angle glaucoma and acute angle-closure glaucoma for over 100 years. In 1994, an oral formulation of pilocarpine (**6**) was approved by the FDA to treat dry mouth (xerostomia) which is a side effect of radiation therapy for head and neck cancers and also used to stimulate sweat glands to measure the concentrations of sodium and chloride (Figure 1) [31]. In 1998, the oral preparation was approved for the management of Sjogren's syndrome, an autoimmune disease that damages the salivary and lacrimal glands.

### 2.1. Natural Products from Fungi

Macro and micro fungi have been part of human life for thousands of years. They were used as food (mushrooms), in preparation of alcoholic beverages (yeasts), medication in traditional medicine and for cultural purposes. Currently with the advances in microbiology, their uses have extended to enzymes, biological control, antibiotics and other pharmacologically active products [32].

**Figure 2:**
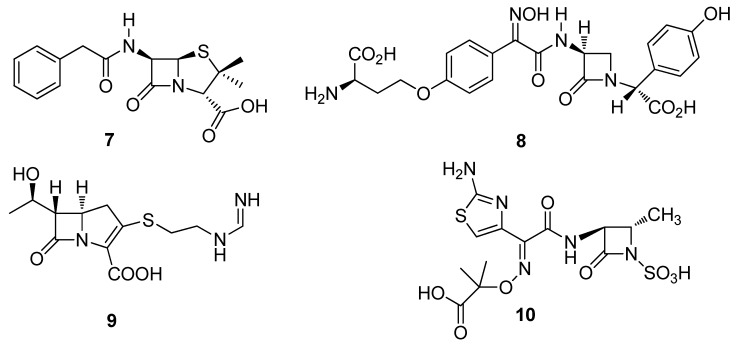
Penicillin (**7**), Norcardicin (**8**), Imipenem (**9**) and Aztreonam (**10**).

Undoubtedly one of the most famous natural product discoveries derived from a fungus (microorganism) is that of penicillin (**7**) from the fungus, *Penicillium notatum* discovered by Fleming in 1929 [33]. A countercurrent extractive separation technique which produced **7** in high yields was required for the *in vivo* experimentation that ultimately saved countless lives and won Chain and Florey (together with Fleming) the 1945 Nobel prize in Physiology and Medicine (Figure 2) [34]. This discovery led to the re-isolation and clinical studies by Chain, Florey and co-workers in the early 1940s and commercialization of synthetic penicillins, which ultimately revolutionized drug discovery research [35,36,37,38].

After publication of the first clinical data on penicillin G (**7**) between 1942–1944 there was a worldwide endeavor to discover new antibiotics from microorganisms and bioactive natural products [39,40]. Up until 1968, old methods for detecting *β*-lactams were still being utilized and it was concluded that all natural *β*-lactams had been discovered [39]. Nevertheless, this was not the case as with the introduction, in the 1970s, of new screening methods, the production of bacterial strains supersensitive to *β*-lactams, tests for the inhibition of *β*-lactamases and specificity for sulphur-containing metabolites resulted in the discovery of novel antibiotic structural classes (norcardicins, carbapenems and monobactams) including the isolation of the antibiotics, norcardicin (**8**), imipenem (**9**) and aztreonam (**10**), respectively (Figure 2) [39,41]. There are presently nine *β*-lactams (two cephalosporins, six carbapenems and one penem) in clinical trials or undergoing drug registration, along with the novel class of broad spectrum antibiotics called the glycylcyclines [41].

Macro fungi such as polypores are a large group of wood-rotting fungi of the phylum Basidiomycota (basidomycetes) and Ascomycota, which are a major source of pharmacologically active substances. There are about 25,000 species of basidiomycetes, of which about 500 are members of the Aphyllophorales [42]. Approximately 75% of tested polypore fungi have shown strong antimicrobial activities and may constitute a viable source for the development of novel antibiotics. Many compounds have displayed antiviral, cytotoxic, antineoplastic, cardiovascular, anti-inflammatory, immune-stimulating and anticancer activities [42,43]. Fungi are more commonly microorganisms, some of which can spend at least part of their life cycle inside plant tissues without causing any visible sign of infection or disease [44,45]. They have been found to inhabit trees, grasses, algae and herbaceous plants and live in the intercellular spaces of plant stems, petioles, roots and leaves without affecting the host organism [46]. Collectively these fungi are known as endophytes. Novel bioactive secondary metabolites derived from fungal sources have yielded some of the most important natural products for the pharmaceutical industry [3].

**Figure 3:**
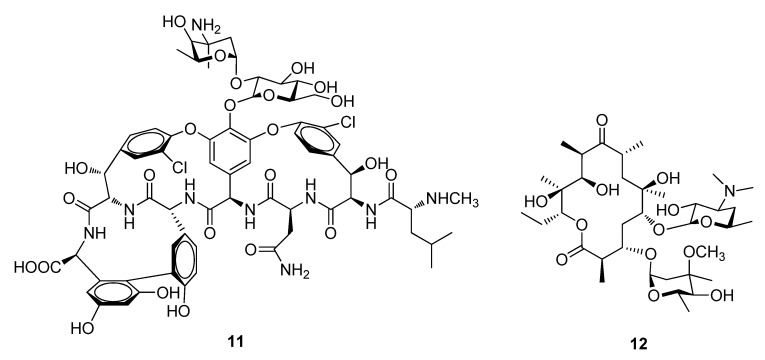
Vancomycin (**11**) and Erythromycin (**12**).

In 1953, Edmund Kornfeld first isolated vancomycin (**11**) a glycopeptide antibiotic produced in cultures of *Amycolatopsis orientalis* which is active against a wide range of gram-positive organisms such as *Staphylococci* and *Streptococci* and against gram-negative bacteria, mycobacteria and fungi and was approved by the FDA in 1958 (Figure 3). It is used for the treatment of severe infection and against susceptible organisms in patients hypersensitive to penicillin (**7**) [5]. The macrolide erythromycin (**12**) from *Saccharopolyspora erythraea* is an antibacterial drug, which contains a 14-membered macrocycle composed entirely of propionate units (Figure 3). Erythromycin (**12**) has broad spectrum activities against gram-positive *cocci* and *bacilli* and is used for mild to moderate, upper and lower respiratory tract infections [5,26]. Currently there are three semisynthetic ketolide derivatives of erythromycin (**12**), cethromycin (ABT-773, Restanza^TM^), EP-420 (by Enanta Pharmaceuticals) and BAL-19403 (by Basilea) which are in clinical development [1].

**Figure 4:**
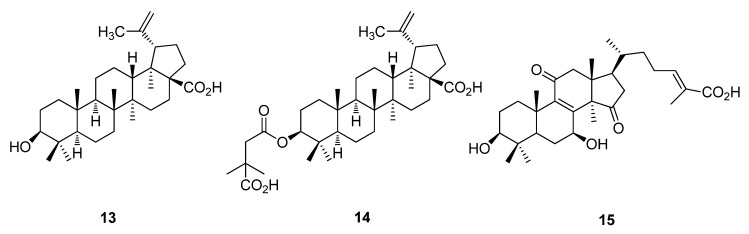
Betulinic acid (**13**), Bevirimat (PA-457) (**14**) and Ganoderic acid *β* (**15**).

Single cell viruses represent the smallest existing life forms causing cold, influenza, ebola and SARS. Presently, there seems to be a limited number of antiviral natural products or synthetically derived analogues from fungi [47]. Betulinic acid (**13**), a triterpenoid obtained from the bark of *Betula pubescens* was originally identified as a weak inhibitor of HIV replication [48,49]. Betulinic acid can inhibit topoisomerase I and is being evaluated in Phase I trials as a cancer chemo-preventive agent (Figure 4) [50]. Bevirimat (PA-457) (**14**), extracted from a Chinese herb *Syzygium claviflorum* is in Phase IIb clinical trials and is believed to inhibit the final step of the HIV Gag protein processing [51]. Ganoderic acid *β* (**15**), isolated from the fruiting bodies and spores of *Ganoderma lucidum*, displayed significant anti-HIV-1 protease activity with an IC_50_ value of 20 µM (Figure 4) [52].

**Figure 5:**
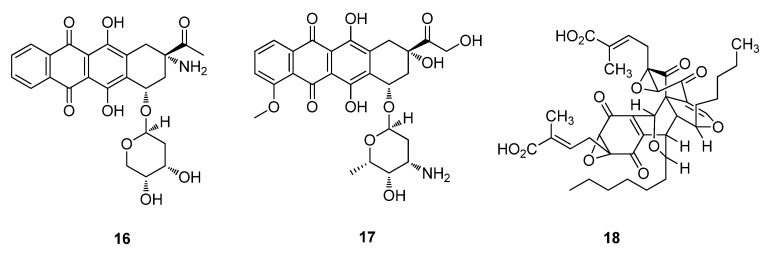
Amrubicin hydrochloride (**16**), Doxorubicin (**17**) and Torreyanic acid (**18**).

In 2002, amrubicin hydrochloride (**16**), related to the anthracycline, doxorubicin (**17**) (Adriamycin®), was isolated from the fungus *Streptomyces peucetius*. Doxorubicin (**17**) is used to treat acute leukaemia, soft tissue and bone sarcomas, lung cancer, thyroid cancer and both Hodgkins and non-Hodgkins lymphomas (Figure 5) [5,26]. Torreyanic acid (**18**) was isolated from an endophyte from the endangered tree, *Torreya taxifolia* [53] and was tested in several cancer cell lines and found to display 5–10 times greater potentency/cytotoxicity in cell lines that are sensitive to protein kinase C causing cell death by apoptosis (Figure 5) [54].

### 2.2. Natural Products from Plants

Plants have been well documented for their medicinal uses for thousands of years. They have evolved and adapted over millions of years to withstand bacteria, insects, fungi and weather to produce unique, structurally diverse secondary metabolites. Their ethnopharmacological properties have been used as a primary source of medicines for early drug discovery [55,56]. According to the World Health Organization (WHO), 80% of people still rely on plant-based traditional medicines for primary health care [57] and 80% of 122 plant derived drugs were related to their original ethnopharmacological purpose [58]. The knowledge associated with traditional medicine (complementary or alternative herbal products) has promoted further investigations of medicinal plants as potential medicines and has led to the isolation of many natural products that have become well known pharmaceuticals.

**Figure 6:**
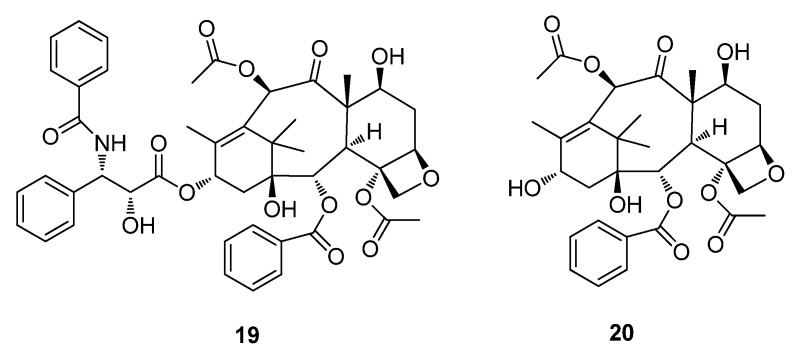
Paclitaxel (Taxol®) (**19**) and baccatin III (**20**).

The most widely used breast cancer drug is paclitaxel (Taxol®) (**19**), isolated from the bark of *Taxus brevifolia* (Pacific Yew). In 1962 the United States Department of Agriculture (USDA) first collected the bark as part of their exploratory plant screening program at the National Cancer Institute (NCI) (Figure 6) [59]. The bark from about three mature 100 year old trees is required to provide 1 gram of **19** given that a course of treatment may need 2 grams of the drug. Current demand for **19** is in the region of 100–200 kg per annum (*i.e.*, 50,000 treatments/year) and is now produced synthetically [26]. The first of several FDA approvals for various uses for Taxol® was announced in 1992 [60]. Taxol® (**19**) is present in limited quantities from natural sources, its synthesis (though challenging and expensive) has been achieved [61]. Baccatin III (**20**) present in much higher quantities and readily available from the needles of *T. brevifolia* and associated derivatives is an example of a structural analogue that can be efficiently transformed into **19** (Figure 6) [26].

**Figure 7:**
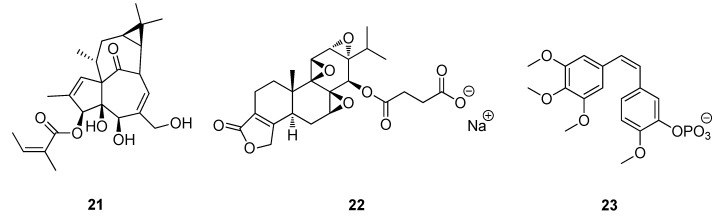
ingenol 3-*O*-angelate (**21**), PG490-88 (**22**) and Combretastatin A-4 phosphate (**23**).

Other examples of antitumor compounds currently in clinical trials include ingenol 3-*O*-angelate (**21**) a derivative of the polyhydroxy diterpenoid ingenol isolated from the sap of *Euphorbia peplus* (known as “*petty spurge*” in England or “*radium weed*” in Australia) which is a potential chemotherapeutic agent for skin cancer is currently under clinical development by Peplin Biotech for the topical treatment of certain skin cancers (Figure 7) [62,63]. PG490-88 (**22**) (14-succinyl triptolide sodium salt), a semisynthetic analogue of triptolide is a diterpene-diepoxide isolated from *Tripterygium wilfordii* which is used for autoimmune and inflammatory diseases in the People’s Republic of China [64,65]. Combretastatin A-4 phosphate (**23**) a stilbene derivative from the South African Bush Willow, *Combretum caffrum* acts as an anti-angiogenic agent causing vascular shutdowns in tumors (necrosis) and is currently in Phase II clinical trials (Figure 7) [66,67].

**Figure 8:**
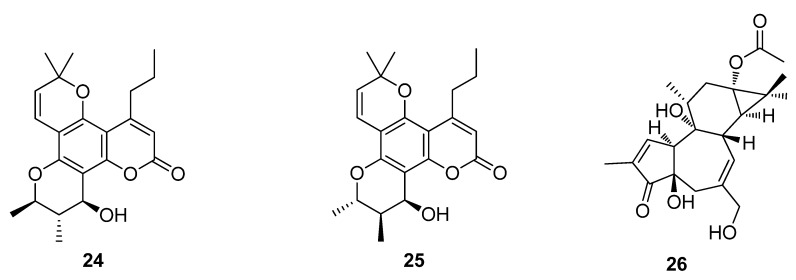
Calanolide A (**24**), Calanolide B (**25**) and Prostratin (**26**).

The Acquired Immune Deficiency Syndrome (AIDS) pandemic in the 1980s forced the National Cancer Institute (NCI) and other organizations to explore natural products as sources of potential drug candidates for the treatment of AIDS. Over 60,000 extracts of plants and marine organisms were tested against lymphoblastic cells infected with HIV-1. The most important result of these tests is the class of compounds known as the calanolides. In particular the isolation of calanolide A (**24**) and calanolide B (**25**) from the *Calonphyllum* species, along with prostratin (**26**) from *Homalanthus nutans*, have now progressed into clinical and preclinical development (Figure 8) [68,69,70]. Calanolide A (**24**) was licensed and evaluated to Phase II clinical trials by Sarawak Medichem Pharmaceuticals, however there has been no subsequent announcement for further drug development. In 2010, Phase I human clinical trials of prostratin (**26**) were carried out by the AIDS ReSearch Alliance in Los Angeles, California (Figure 8).

**Figure 9:**
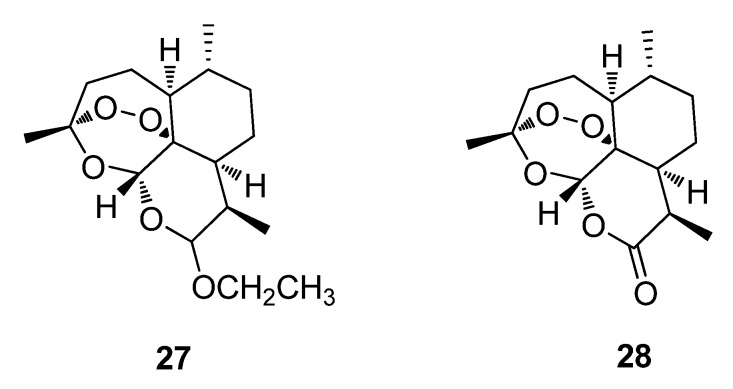
Arteether (**27**) and artemisinin (**28**).

Arteether (**27**), introduced in 2000, as Artemotil is derived from artemisinin (**28**) (introduced in 1987 as Artemisin) which was first isolated from the plant *Artemisia annua* and are both approved antimalarial drugs (Figure 9) [47]. The plant was originally used in traditional Chinese medicine as a remedy for chills and fevers. Other derivatives of artemisinin (**28**) are in various stages of clinical development as antimalarial drugs in Europe [3,26]. To date, a synthetic trioxolane modeled on the **28** pharmacophore, is being assessed in combination with piperaquine (a synthetic bisquinoline antimalarial drug) in an effort to treat malaria (Figure 9) [71].

**Figure 10:**
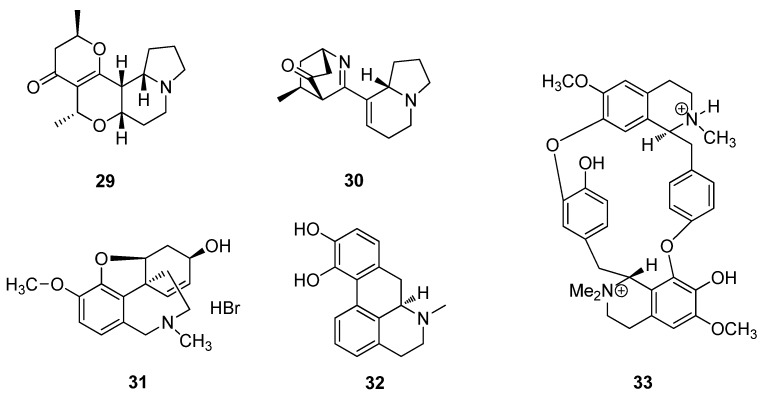
Grandisine A (**29**), Grandisine B (**30**), morphine (**31**), apomorphine (**32**) and tubocaurarine (**33**).

Grandisines A (**29**) and B (**30**) are two indole alkaloids which were isolated from the leaves of the Australian rainforest tree, *Elaeocarpus grandis* (Figure 10). Grandisine A (**29**) contains a unique tetracyclic skeleton, while Grandisine B (**30**) possesses an unusual combination of isoquinuclidinone and indolizidine ring systems. Both **29** and **30** exhibit binding affinity for the human *δ*-opioid receptor and are potential leads for analgesic agents [72]. Galantamine hydrobromide (**31**) is an Amaryllidaceae alkaloid obtained from the plant *Galanthus nivalis* and has been used traditionally in Turkey and Bulgaria for neurological conditions and is used for the treatment of Alzheimer’s disease [73,74]. Apomorphine (**32**) is a derivative of morphine (**31**) isolated from the poppy (*P. somniferum*) and is a short-acting dopamine *D_1_* and *D_2_* receptor agonist, as well as a potent dopamine agonist, used to treat Parkinson’s disease (Figure 10) [75]. *“Curare”* is the arrow poison of the South American Indians and is prepared in the rain forests of the Amazon and Orinoco. Tubocaurarine (**33**) isolated from the climbing plant, *Chondrodendron tomentosum* (Menispermaceae) is one of the active constituents used as a muscle relaxant in surgical operations, reducing the need for deep anesthesia. The limited availability of tubocurarine (**33**) has led to the development of a series of synthetic analogues which are now preferred over the natural product (Figure 10) [26].

### 2.3. Natural Products from the Marine Environment

Though plants have proven to be a novel source for bioactive natural products the marine environment has a clear track record in also offering novel structural entities. “*We are not marine organisms*”, says Fenical, “*so until about 1970, no one even thought of the ocean. It was left as a deep secret. It seemed ridiculous to me that the ocean — with such a vast habitat — had escaped anyone's notice. But there are good reasons. People fear the ocean; it has been considered a very hostile, inhospitable place*” [76]. Given that 70% of planet earth’s surface is covered by ocean, pharmaceutical companies began to realize that the ocean would possess unique biodiversity and may be a possible source for potential drug candidates [77].

Exploration of the marine environment and organisms (algae, sponges, ascidians, tunicates and bryozoans) became possible due to modern snorkeling, the introduction of SCUBA (1970s), to the use of manned submersibles (1980s) and more recently the use of remotely operated vehicles (ROVs) (1990s). These progressive advancements in the past 40 years of exploration of the marine environment have resulted in the isolation of thousands of structurally unique bioactive marine natural products. To date, the global marine pharmaceutical pipeline consists of three Food and Drug Administration (FDA) approved drugs, one EU registered drug, 13 natural products (or derivatives thereof) in different phases of the clinical pipeline and a large number of marine chemicals in the pre-clinical pipeline [78]. Some examples include, Ziconotide (Prialt®, Elan Corporation) a peptide first discovered in a tropical cone snail, which was approved in December 2004 for the treatment of pain.

**Figure 11:**
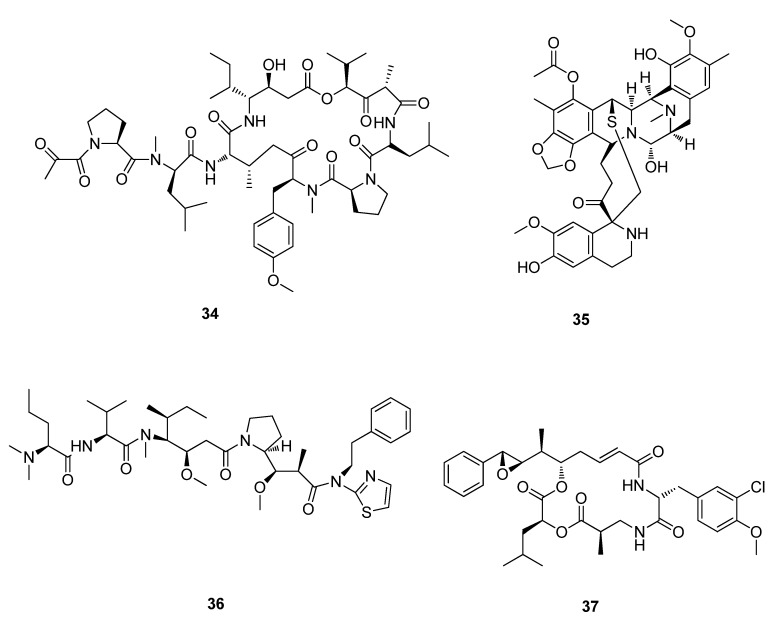
Plitidepsin (**34**), ET743, Spisulosine (**36**) and Cryptophycin (**37**).

Plitidepsin (**34**) (Aplidin®, PharmaMa), a depsipeptide was isolated from the Mediterranean tunicate *Aplidium albicans* [79,80]. Plitidepsin (**34**) is effective in treating various cancers, including melanoma, small cell and non-small cell lung, bladder as well as non-Hodgkin lymphoma and acute lymphoblastic leukemia and is currently in Phase II clinical trials (Figure 11) [78,81]. Ecteinascidin 743 (ET743; Yondelis™) (**35**) was isolated in very low yields from the ascidian *Ecteinascidia turbinata* [82,83]. The quantities of ET743 (**35**) required for advanced pre-clinical and clinical studies was achieved by adopting very large-scale aquaculture of *E. turbinata* in open ponds, however the semisynthesis of ET743 (**35**) has been well established (Figure 11) [81,84,85]. In October 2007, Trabectedin (**35**) (also known as Ecteinascidin 743 or ET-743) (Yondelis, PharmaMar) became the first marine anticancer drug to be approved in the European Union [78]. Trabectedin (**35**) has been approved by the European Agency for the Evaluation of Medicinal Products (EMEA) and is completing key Phase III studies in the US for approval [78]. Spisulosine (**36**), isolated from the marine clam *Spisula polynyma*, exhibited substantial selective activity against tumor cells compared to normal cells [86]. It advanced to Phase I clinical trials against solid tumors but was withdrawn in late 2006 [87,88,89]. Cryptophycin (**37**) was selected for clinical trials in the mid 1990s then advanced to phase II trials but was terminated in 2002 due to toxicity and lack of efficacy (Figure 11) [90].

### 2.4. Natural Products from Marine Algae

Algae (macroalgae, seaweed) are represented by at least 30,000 species worldwide supplying oxygen to the biosphere, food for fish and man, medicine and fertilizers as well as being a prolific source of structurally unique natural products [91]. The terpenoids are a class of compounds predominantly isolated from marine algae in the 1970–1980s. Chemical investigations into terpenoid-type structures have led to the isolation of many classes including brominated, nitrogen and oxygen heterocycles, phenazine derivatives, sterols, amino acids, amines and guanidine derivatives [92]. With respect to biological activity, green, brown and red algae have been intensively assessed for their antibacterial and antifungal activities [93].

**Figure 12:**
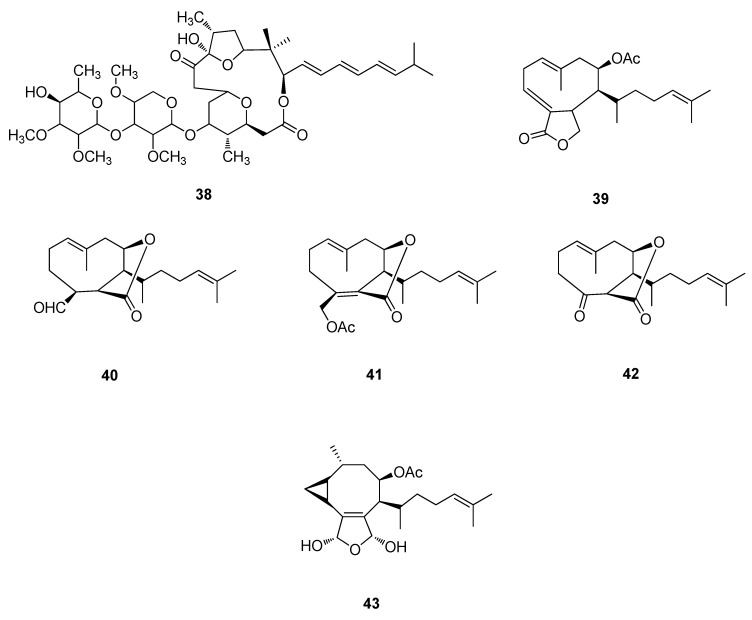
Polycavernoside-A (**38**), 4-acetoxydictylolactone (**39**), Dictyolide A (**40**), Dictyolide B (**41**), Nordictyolide (**42**) and Crenuladial (**43**).

Polycavernoside-A (**38**) isolated from the red alga *Polycaverosa tsudai* was suspected to be the toxic glycoside responsible for seafood poisoning, when, in 1991 thirteen people became ill and three died in Japan (Figure 12) [94,95,96]. The brown alga, *Dictyota dichotoma* afforded diterpenes, 4-acetoxydictylolactone (**39**), dictyolides A (**40**), B (**41**) and nordictyolide (**42**) which display antitumor activities [97,98]. Another example is crenuladial (**43**), isolated from the brown alga *Dilophus ligatus* which displayed antimicrobial activity against *Staphylcoccus aureus*, *Micrococcus luteus* and *Aeromonas hydrophyla* (Figure 12) [98,99].

**Figure 13:**
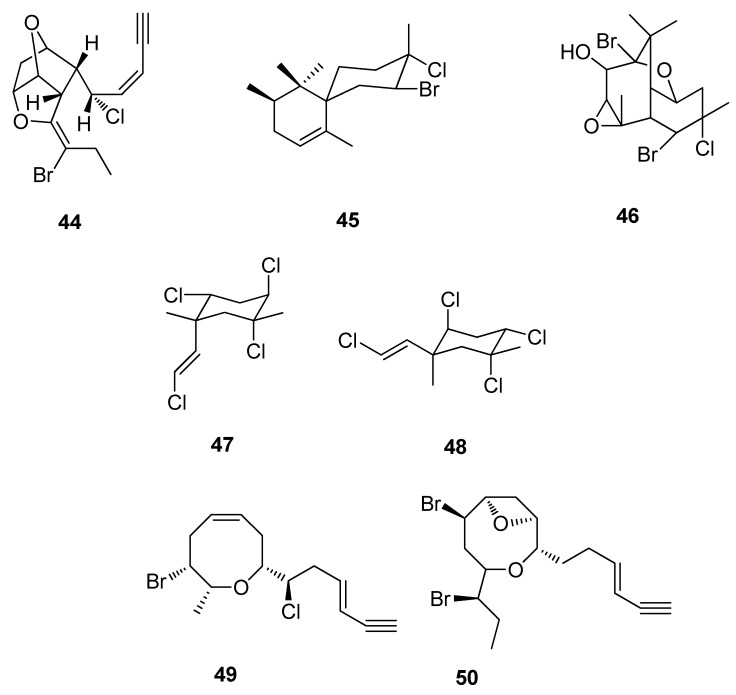
Compound (**44**), Compound (**45**), Compound (**46**), 1*α*-(2-*E*-chlorovinyl)-2*α*,4*β*,5*α*-trichloro-1*β*,5*β*-dimethylcyclohexane (**47**), 1*β*-(2-*E*-chlorovinyl)-2*β*,4*α*,5*α*-trichloro-1*α*,5*β*-dimethylcyclohexane (**48**), Laurepinnacin (**49**) and (*Z*)-laureatin (**50**).

Red algae, in particular the genus *Laurencia* (Rhodophyta), are unsurpassed as a source of halogenated sesquiterpenes. Chemical investigations into the genus *Laurencia* for secondary metabolites have been active since the 1970s. The most commonly occurring secondary metabolites are the halogenated sesquiterpenes and diterpenes. Furthermore, this genus is unique in producing C_15_-acetogenins, for example those constituents which possess a terminal enyne such as **44** [100]. Other examples include the class of compounds known as the chamigrenes, which are halogenated terpenes possessing unique structures such as **45** and **46** (Figure 13). There have been many chamigrenes, which have been isolated to date from the genus *Laurencia*, which grows in many very different geographical areas [101,102,103,104].

Productivity in agriculture in the last half century has been as a result of advances in pest control due to synthetic chemical pesticides (SCPs) [105]. However, the search for new pesticides has been necessary due to the significant rise in the resistance to current control agents. It has been documented that between 1984 to 1990 resistance to synthetic chemical pesticides by insects and mites increased by 13% [106,107]. As a result, a significant amount of research has focused on the isolation of insecticidal leads from marine algae. This has led to the isolation of over 40 active constituents [107]. Some examples of natural insecticides include the isolation of 1*α*-(2-*E*-chlorovinyl)-2*α*,4*β*,5*α*-trichloro-1*β*,5*β*-dimethylcyclohexane (**47**) and 1*β*-(2-*E*-chlorovinyl)-2*β*,4*α*,5*α*-trichloro-1*α*,5*β*-dimethylcyclohexane (**48**) which are cyclic polyhalogenated monoterpenes isolated from the Chilean red alga *Plocamium cartilagineum* (Figure 13). These compounds show insecticidal activity against the Aster leafhopper, *Macrosteles fascifrons* [108]. Other examples include laurepinnacin (**49**), an acetylenic cyclic ether from the red alga *Laurencia pinnata* Yamada [109], and (*Z*)-laureatin (**50**) and related compounds from the red alga *L. nipponica* Yamada. These have all shown to display potent insecticidal activity against the mosquito larva, *C. pipiens* (Figure 13) [110].

### 2.5. Natural Products from Marine Sponges

**Figure 14:**
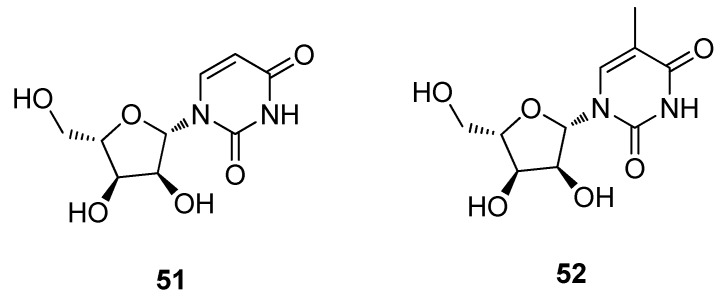
Spongouridine (**51**) and Spongothymidine (**52**).

Sponges (Porifera) are sessile organisms, which lack a nervous, digestive and circulatory system and maintain a constant water flow through their bodies to obtain food, oxygen and to remove wastes. All sponges are ‘current’ or ‘filter’ feeders and have few physical means of defense against predators. They are considered to be the first multicellular animals and have changed very little in approximately 500 million years. The first notable discovery of biologically active compounds from marine sources can be traced back to the reports of Bergmann on the isolation and identification of *C*-nucleosides, spongouridine (**51**) and spongothymidine (**52**) from the Caribbean sponge, *Cryptotheca crypta* in the early 1950s (Figure 14) [111]. These compounds were found to possess antiviral activity and the synthesis of structural analogues led to the development of cytosine arabinoside (Ara-C) as a clinical anticancer agent, together with (Ara-A) as an antiviral agent 15 years later [111]. This was an important discovery since previously it was believed that for a nucleoside to possess biological activity, it had to have a deoxyribose or ribose sugar moiety. These investigations led to the identification of (Ara-C) as a potent antileukemic agent [112].

### 2.6. Natural Products from other Marine Sources

**Figure 15:**
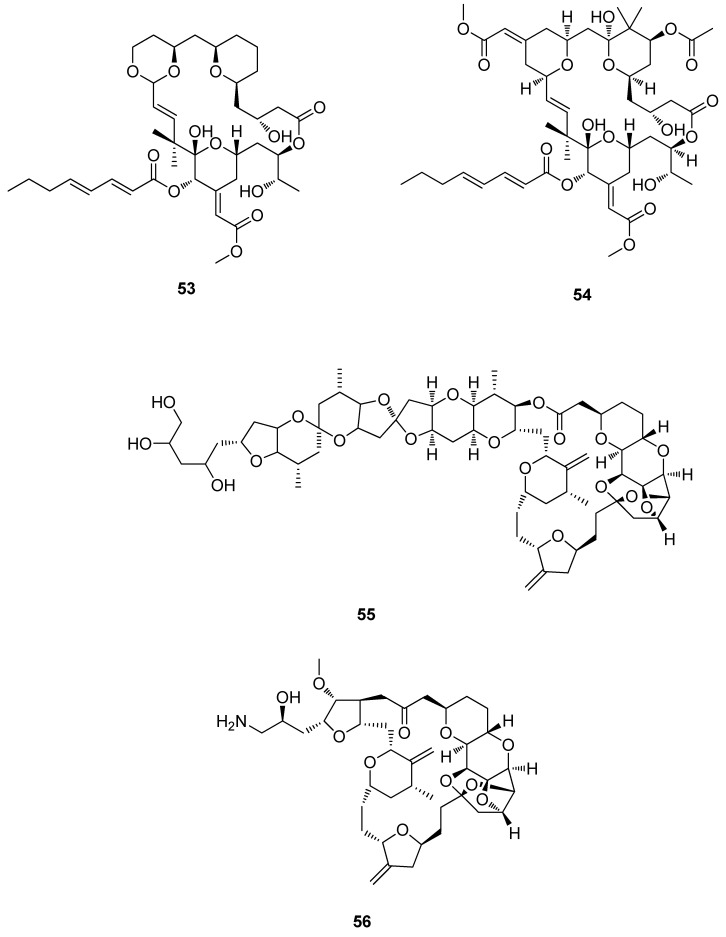
Compound (**53**), Bryostatin 1 (**54**) Halichondrin B (**55**) and Halichondrin E-7389 (**56**).

The class of synthetic derivatives known as the bryologs, such as **53**, are derived from bryostatin 1 (**54**), an antineoplastic compound isolated from the bryozoan, *Bulgula neritina* [5,113]. Bryostatin 1 (**54**) has been isolated in sufficient quantities to permit more than 80 clinical trials to date, with 20 being completed at both phase I and phase II levels (Figure 15) [78]. It has displayed positive responses acting as a single agent with effects ranging from complete to partial remission [28]. From 2007 to date there were four Phase I and eight Phase II clinical trials, all combination studies with biologicals or cytotoxins against multiple carcinomas. Currently, **54** is in two Phase I clinical trials and is being assessed as an anti-Alzheimer’s drug (Phase I trial approved) [78]. Halichondrin B (**55**) has been isolated from several sponges including, *Halichondria okadai* (Japan) [114]; *Axinella* sp. from the Western Pacific [115], *Phakellia carteri* from the Eastern Indian Ocean [116] and from *Lissodendoryx* sp., off the East Coast of the South Island of New Zealand (Figure 15) [117]. Halichondrin B (**55**) has been successfully synthesized [118] along with several structural analogues including Halichondrin E-7389 (**56**) which has been selected for further development and is currently in phase III clinical trials for the treatment of breast carcinoma (Figure 15) [113].

## 3. Drug Discovery: Natural Product Chemistry *versus* Combinatorial Chemistry

**Figure 16:**
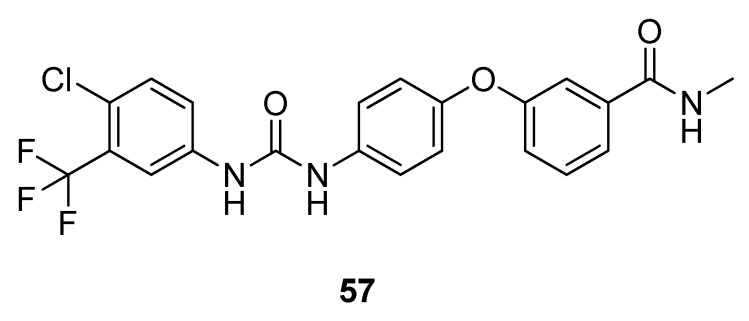
Sorafendib (**57**).

After the *‘Golden Age of Antibiotics’* and the worldwide incentive to discover new antibiotics many major pharmaceutical industries at the time initiated natural product discovery (NPD) programs which focused not only on antibacterial and antifungal targets but also on infectious diseases. These programs provided lead compounds for the treatment of cancer, microbial infections, hypercholesteremia and tissue rejection in organ transplantations [119,120]. However, many of the larger pharmaceutical companies decommissioned their NPD programs during the 1990s and early 2000s. It was the advent of automated high throughput screening (HTS) which increased the momentum of biological testing and combinatorial chemistry began to be promoted as a better approach to creating “*drug-like*” compounds for HTS. As a result, many of the pharmaceutical companies disbanded or sold their collections of screening extracts [121,122] as it was believed that traditional extract-based screening resulted in the continuous re-discovery of previously isolated compounds and that the structural complexity of natural products required total synthesis and derivatization which is both economically and synthetically problematic. Because of supply problems, the time required to develop a natural product from an extract hit to a pharmaceutical was deemed to be too long; HTS technologies rely on combinatorial chemistry to generate large compound libraries. In the past two decades “*classical natural product chemistry*” has largely been replaced by molecular target based drug discovery, utilizing large combinatorial libraries to obtain efficient “*hits*” [120]. Nevertheless advances in technology and sensitive instrumentation for the rapid identification of novel bioactive natural products and structure elucidation continues to improve the natural product discovery process [119]. From the 1980s onwards it was thought that combinatorial chemistry would be the future source of numerous novel carbon skeletons and drug leads or new chemical entities (NCEs). This has clearly not been the case as there has only been one combinatorial NCE approved by the U.S Food and Drug Administration (FDA) in that time period, the kinase inhibitor sorafendib (**57**) (approved by the FDA, 2005) for renal carcinoma (Figure 16) [123].

Combinatorial chemistry has indeed revolutionized the development of novel active chemical leads resulting in the synthesis of structural analogues [123]. At the time, combinatorial libraries consisted of hundreds to thousands of new compounds, but during the late 1990s synthetic chemists realized that these libraries lacked the complexity of the intricate natural products synthesized by nature [123]. The concept of diversity-oriented synthesis (DOS) was adopted in which synthetic chemists would synthesize compounds that resembled natural products (mimics) or that are based on natural product topologies. These compounds are currently being tested in a large number and variety of biological screens in order to determine their role (s) as leads to novel drug entities [123]. Inspection of the rate of NCEs approvals demonstrates that natural products still contribute to or are involved in ~50% of all small molecule testing between the years 2000–2006 [47]. Though the pharmaceutical industry has expended a considerable amount of resources to both HTS and combinatorial chemistry [47] overall of the 1184 NCEs covering all diseases/countries/sources between the years 1981–2006, 30% were found to be synthetic. It is also worth noting that 52% (total) of these compounds are either a natural product, a mimic or a chemical modification of an existing natural product pharmacophore [47].

### 3.1. Dereplication

A drug discovery program endeavors to search for (a) novel bioactive natural product(s), which possess(es) some form of potent biological activity. Nevertheless, the isolation of known and undesirable natural products with no chemical or pharmacological interest is inevitable. The process of identifying known compounds responsible for the activity of an extract prior to bioassay-guided isolation is referred to as dereplication [28,29]. At present there are many advanced methodologies and protocols that distinguish novel entities from known natural compounds at an early stage of a drug discovery program or in a natural product isolation strategy [29]. It is important to realize that the isolation of novel natural products was far more frequent during the 1970s and is steadily declining, although natural sources (e.g., plants, fungi, marine and microbial sources) are still regarded as inexhaustible sources for novel chemicals. As such, the time, effort and cost to find new chemical entities must be considered as their discovery has become far more infrequent [124].

Furthermore, even the selection of new types of organisms (terrestrial and/or marine) tends to lead to the rediscovery of previously reported natural products as they are often present in more than one phylum or genera [125]. It has been estimated that it takes $US 50,000 and three months of work to isolate and identify an active compound from a natural product source [126]. Therefore, it is exceedingly important to recognize previously known compounds early on, not only for saving time and money, but to allocate resources to more profitable extracts. It is evident that natural product programs require more patience and perseverance for the identification of adequate lead compounds than programs strictly based on synthetic chemicals. This is also dependent on availability of bioassay-guided fractionation, *in*-house screening, accessibility to higher field NMR and mass spectrometers, all of which are necessary to efficiently run such a program. Lead compounds arising from natural product discovery programs are structurally unique due to their co-evolution with target sites in biological systems. However, the speed at which lead compounds can be generated and progressively advanced is slower than corresponding synthetic drug discovery approaches [119]. With the advent of new hyphenated spectroscopy technologies such as HPLC-MS, HPLC-NMR and HPLC-NMR-MS, further means of rapid compound identification and dereplication are now possible [119,127].

### 3.2. Dereplication Methods

Dereplication strategies generally involve a combination of bioassay, separation science, spectroscopic methods, and database searching and can be regarded as chemical or biological screening processes. There are a number of ways in which natural product programs approach dereplication, which is based upon availability of screening methods/instrumentation, time and the cost to identify possible “*biological leads or novel compounds*” from a crude extract. 

### 3.3. Database Searching

There are many commercially available databases, which can assist in the dereplication process and will often reduce the time taken for structure elucidation of known compounds. The Chapman and Hall Dictionary of Natural Products [128]; The Dictionary of Marine Natural Products (on-line) (subset of the Dictionary of Natural Products) containing over 30,000 compounds [129]; MarinLit- The Marine Natural Products Database containing up to date bibliographic data on marine organisms with the number of references from 1,200 journals/books and data for ~21,000 compounds [130]; AntiMarin is a more recent database, in which the number of methyl groups, the number of *sp^3^*-hybridised methylene or methine protons, alkene, acetal, ether and formyl groups can be searched [131,132]. SciFinder Scholar and SCOPUS is a research discovery tool (Chemical Abstracts on-line) [133,134] and NAPRALERT^TM^ is a database of all natural products, including ethnomedical information, pharmacological/biochemical information of extracts of organisms *in vitro*, *in situ*, *in vivo*, in humans (case reports, non-clinical trials) and clinical studies [135]. Access to scientific databases such as the ones mentioned, is a fundamental and crucial step in a well-governed natural product program. Thorough and extensive literature searches are necessary when the following questions need to be addressed:

- Have there been any previous literature reports on the target organism (terrestrial or marine?)
- Is there potential to isolate novel compounds (geographical or seasonal variations?)
- What kind of compound classes has been isolated from the species and if not from the species, then the genus or family?
- Is there incomplete or poor NMR spectroscopic data for previously uncharacterized natural products?
- Are there any new biological activities for known compounds that have been overlooked?

It is fundamentally important to address these questions early as one of the most common issues that occurs is the time consuming process of isolating, purifying and determining the structure of a suspected novel compound and realizing that it has already been reported in the literature.

### 3.4. Hyphenated Instrumentation “Classical versus Hyphenated (on-line) Approaches”

Natural product extracts often contain a large number of constituents including those, which are challenging to separate. The combination of classical techniques such as Ultraviolet absorption Spectroscopy (UV), Infra-red spectroscopy (IR), Mass spectrometry (MS) and Nuclear Magnetic Resonance spectroscopy (NMR) often permits the unambiguous structure determination of pure compounds. In cases where the absolute configuration cannot be determined, synthesis or single-crystal X-ray analysis is utilized. As classical separation techniques are tedious and time consuming, the direct hyphenation of an efficient separation technique with powerful spectroscopic techniques can assist in the dereplication process [136]. Such hyphenated systems (though not in widespread use) include HPLC-FTIR, which is useful for the detection of functional groups in major constituents of mixtures. HPLC-FTIR has been used by their designers but has not found wide application due to limitations in compatibility; that is, obtaining optimal separation together with sufficient detection [136].

FIA-NMR involves a sample, which is injected as a plug into a fluid stream, which is then swept into the NMR detector coil. FIA-NMR uses the mobile phase as a hydraulic-push solvent to carry the injected sample from the injector port to the NMR flow cell. After the pump stops, the spectrometer acquires the scout scan to determine the location of solvent peaks and then acquires the solvent suppressed spectrum. After completion, a signal is sent to the solvent pump to flush the old sample from the NMR flow-cell [136].

HPLC-NMR-MS is an advanced spectrometric hyphenated technique which is used in the dereplication of natural product extracts (typically plant extracts) [137]. Apart from its efficiency, the most important advantage of HPLC-NMR-MS is the unequivocal matching of the MS data to the NMR spectrum. Furthermore, as HPLC-NMR does not provide information about silent functional groups (e.g., hydroxyl and amino moieties) as a result of D_2_O exchange, these functionalities can be readily detected by MS techniques.

Chemical and biological investigation for the search of novel bioactive natural products involves the extraction, isolation, purification and structure elucidation (classical natural product isolation methodologies), which can be challenging and/or time consuming. The extraction is normally the first step for both marine and terrestrial organisms. The choice of the extraction solvent followed by solvent partitioning or by trituration can result in many problems including the formation of artifacts. Further, homogenization and lyophilization with organic solvents can affect the nature and relative amounts of extracted secondary metabolites present. The application of HPLC-NMR to the crude extracts (NMR and UV profile from PDA HPLC detection) was found to be a powerful spectroscopic tool which had advanced in the last decade, in particular with the advent of higher field magnets and cryo-probes. In recent years the advances in microcoil HPLC-NMR and capillary NMR (CapNMR) has allowed for smaller quantities of samples to be analyzed in the order of 40–120 µL, this in combination with higher field magnets has greatly increased the sensitivity in profiling and dereplication natural product extracts [138,139,140]. Microcoil HPLC-NMR is generally suited for on-line HPLC-NMR typically where components present in higher concentrations in an extract are separated and analyzed in a conventional HPLC-NMR system using either on-flow, stop-flow or time slicing experiments [103,141,142,143]. Capillary NMR allows for the use of non-deuterated solvents in the off-line HPLC separation providing a broader range of solvents to be used and low costs. Isolated compounds are redissolved in deuterated solvents and injected into the CapNMR flow probe using volumes of approximately 6 µL with ^1^H-NMR spectra obtained for sample quantities in the order of 2–30 µg, thereby providing a substantial increase in sensitivity with the potential of identifying novel low level secondary metabolites [144].

Apart from providing a *“high-fidelity”* snapshot of the constituents in the extract, the information acquired from both 1D and 2D NMR spectra may be sufficient to identify compound classes, therefore providing information which will allow rational decisions about the best method of fractionation or whether to further pursue the isolation. A number of recent publications have been reported in utilizing this approach [103,104,141,142]. The technique and use of HPLC-NMR in natural products identification/characterization is well documented in the literature but applications of its uses have predominately dealt only with the chemical profiling of plants [145,146,147]. Various modes of HPLC-NMR (predominantly on-flow and stop-flow modes) combine the resolving power of chromatography, which is interfaced with the structural insight provided by NMR.

## 4. Combining Natural Product Chemistry and Metabolomics Approaches in Drug Discovery

Systems biology is an emerging field encompassing tools in the post-genomics revolution such as transcriptomics, proteomics, glycomics and fluxomics with the ambition to characterize all gene and cell products including mRNA, proteins, glycan structures and metabolites in the most comprehensive manner. The objectives of metabolomics are to construct unbiased observations with highly reproducible analytical tools followed by data analysis to locate correlations between all available data. In the emerging field of metabolomics a single analytical technique capable of profiling all low molecular weight metabolites of a given organism does not exist. This emerging field combines analytical chemistry, biochemistry and sophisticated informatics allowing the analysis of thousands of small molecules (metabolites) in any biological system. Mass spectrometry hyphenated with gas chromatography (GC), liquid chromatography (LC) or capillary electrophoresis (CE) and nuclear magnetic resonance (NMR) spectroscopy are the leading analytical platforms. Both primary and secondary metabolites in tissues and biofluids are extracted utilizing unbiased crude extraction procedures aiming to efficiently extract all or most metabolites in their natural form prior to analysis in the solvents used. Since metabolite extracts are extremely complex, given the huge chemical diversity of metabolites they represent, there is no one single analytical platform and methodology, which is capable of analyzing all metabolites simultaneously. Multiple separation chemistries have to be employed to achieve the greatest comprehensiveness in the analysis [148].

Due to the improvement in sensitivity, resolution and advances in instrumentation hundreds of compounds can be simultaneously analyzed with subsequent refined informatics tools developed to extract information from the resulting data, filtering algorithms to remove background noise, detection and integration of peaks throughout large data sets and normalization and transformation of resulting data matrices prior to any statistical analysis can now be achieved [149]. The greatest bottleneck in metabolomics is the ability to identify the detected signals with respect to their chemical nature. Even now, about 60 to 80 % of all detected compounds are unknown [149,150] and the metabolomic community has implemented many different initiatives to tackle the problem by creating large mass spectral or NMR spectral libraries. Many of these unknown structures (*i.e.*, secondary metabolites) detected may be undiscovered natural product resources.

Fingerprinting, footprinting, profiling or target analyses are common terms used in this field. Fingerprinting aims to take a “*snapshot*” of the organism where the signals cannot necessarily be used to detect/identify specific metabolites and depends strongly on the technique used. Metabolite profiling techniques require that signals can be assigned to a specific metabolite whether it is of a known or novel nature. The term target analysis aims to determine and quantify a specific metabolite of interest [151].

There are few reports in the scientific literature, which discuss the unison of classical natural product chemistry approaches with metabolomics to identify novel bioactive natural products. These have generally focused on the study of plants [151]. The identification of bioactive natural products from plants remains a multifaceted task because of their high chemical diversity and complexity. By measuring the metabolome of different extracts or fractions of a plant and combining these data with its corresponding biological activity, signals related to the compounds related to the displayed activity can potentially be determined.

**Figure 17:**
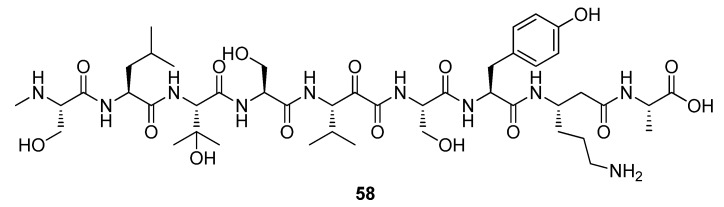
Myxoprincomide (**58**).

In one example, myxoprincomide (**58**), a novel NRPS/PKS natural product from *Myxococcus xanthus* DK1622 was identified by combining methods of targeted mutagenesis, liquid chromatography coupled to high resolution mass spectrometry (LC-HRMS), and a statistical data evaluation (Figure 17). Mutant and wildtype strains were grown in small-scale fermentation in quadruplicate, replicate extracts were analyzed by LCHRMS, and data were pretreated by using a compound finding algorithm, resulting in the definition of >1,000 molecular features per sample. Molecular features specifically missing in culture extracts from DK1622 mutant strains were identified using PCA to the preprocessed LC-MS datasets [152].

**Figure 18:**
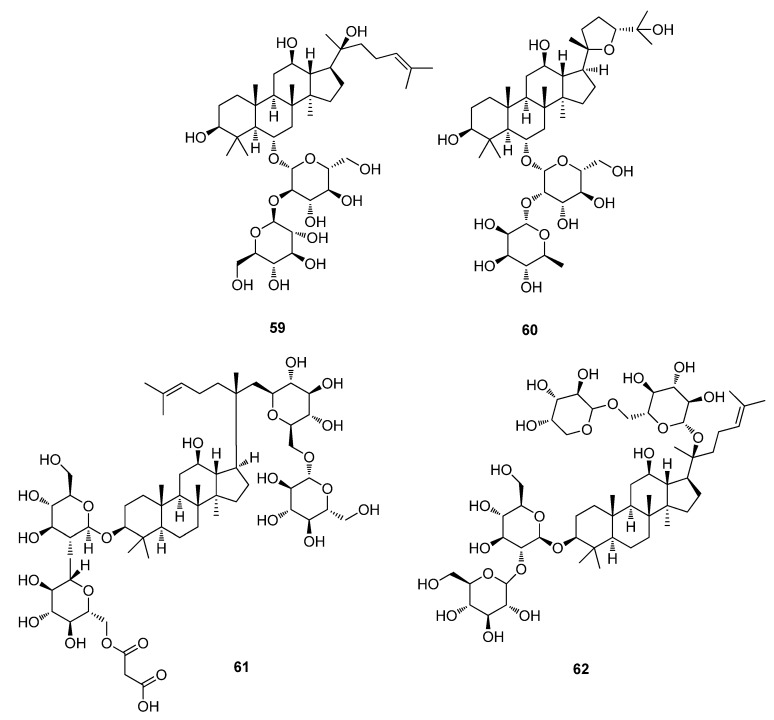
Ginsenoside Rf (**59**), 20(*S*)-pseudoginsenoside F11 (**60**), Malonyl gisenoside Rb1 (**61**), and gisenoside Rb2 (**62**).

Five medicinal *Panax* herbs (ginseng species) were subjected to a metabolomic analysis using Ultra-Performance Liquid Chromatography-Quadrupole Time Of Flight Mass Spectrometry (UPLC-QTOFMS) and multivariate statistical analysis techniques. PCA of the analytical data explained that the five *Panax* herbs could be separated into five different groups of phytochemicals [153]. PCA identified, ginsenoside Rf (**59**), 20(*S*)-pseudoginsenoside F11 (**60**), malonyl gisenoside Rb1 (**61**), and gisenoside Rb2 (**62**) which accounted for the variance which was identified through the loadings plot of the PCA, and tentatively by the accurate mass of TOFMS (Figure 18) [153]. The results and methodology demonstrated this method to be reliable for the rapid analysis of a group of metabolites present in natural products extracts [153].

In the case of NMR of crude extracts, patterns can be visualized and interpreted which is generally combined with multivariate data analysis. This can be carried out in a comparative manner distinguishing differences between relatively similar extracts or it can be linked with a specific (generally *in vitro*) biological activity. Ultimately this enables the construction of a complex database of the metabolome [154,155,156].

**Figure 19:**
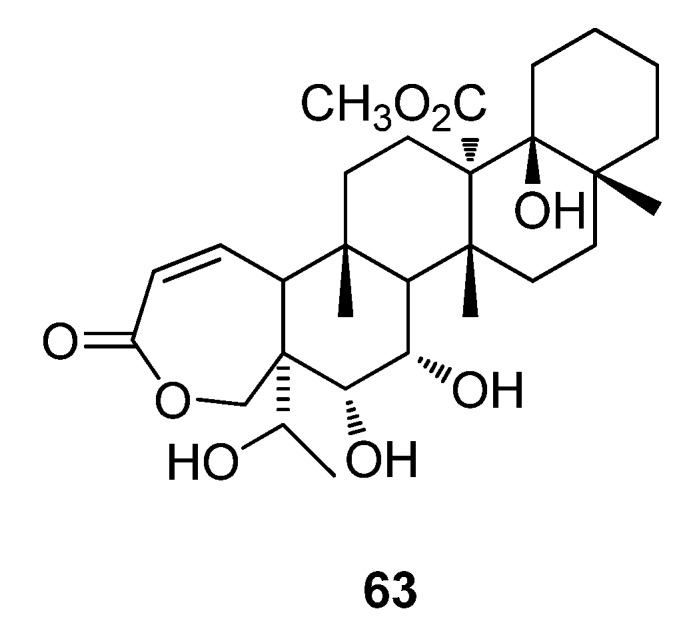
Galphimine (**63**).

An NMR based metabolomics approach has recently been utilized in the study of *Galphimia glauca*, a Mexican plant which has been used in traditional medicine for the treatment of central nervous disorders [157]. Six collections from the Mexican area demonstrated sedative and anxiolytic activities, with only two collections of *G. glauca* showing significant activity. ^1^H-NMR metabolomic profiling was conducted on all six extracts and was analyzed by partial least square-discriminant analysis (PLS-DA) using previous information on their bioactivities [157]. The PLS-DA loadings plot demonstrated a signal strongly correlated with sedative and anxiolytic activities was found to be galphimine (**63**) (Figure 19). A targeted HPLC metabolomic approach was also conducted which provided evidence that the two collections possessing strong sedative and anxiolytic activities contained high amounts of galphimine while both other (less active) samples did not [157].

**Figure 20:**
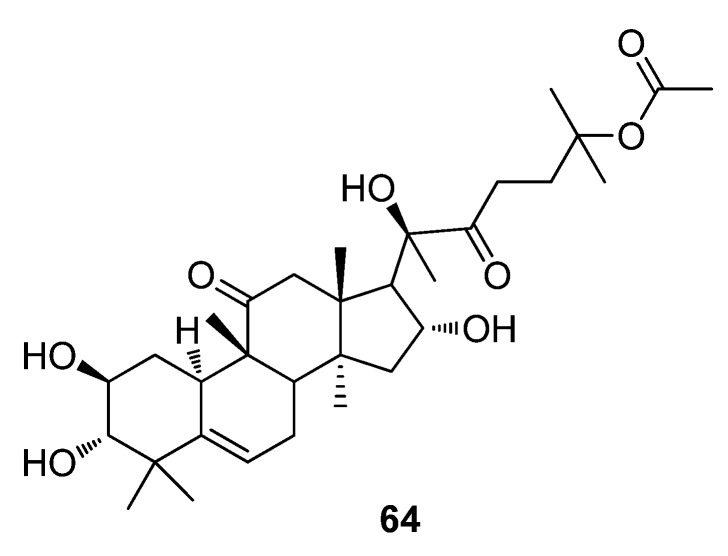
Dihydrocucurbitacin F-25-*O*-acetate (**64**).

Zhi and co-workers analyzed the effect of different antibiotics with different modes of action on various microbes [158]. The results concluded that dihydrocucurbitacin F-25-O-acetate (**64**), a major constituent of the Chinese plant *Hemsleya pengxianensis*, showed antimicrobial activity [158]. The metabolome of a *Staphylococcus aureus* culture treated with a plant extract, **64** and several known antibiotics were compared. PCA analysis revealed that **64** was the component responsible for the main antimicrobial activity on *S. aureus* in *H. pengxianensis* through its ability to inhibit cell wall synthesis, as in the case of vancomycin (Figure 20) [158].

NMR based metabolomics has many applications in plant science and can be used in functional genomics to differentiate plants from different origin, or after different treatments [159]. Kim and co-workers describe the advantage of a NMR metabolomic analysis and the possibility of identifying metabolites by comparing NMR data with references or by structure elucidation using 2D-NMR [159]. Deyrupa and co-workers also demonstrated the use of 2D-NMR spectroscopy to screen a library of biorationally selected insect metabolite samples for partial structures. This investigation enabled the detection of novel compounds in complex metabolite mixtures without prior fractionation or isolation. This led to the discovery and isolation of two families of tricyclic pyrones in *Delphastus catalinae*, a tiny ladybird beetle that is employed commercially as a biological pest control agent [160]. The *D. catalinae* pyrones represent ring systems not previously found in nature [160]*.*

## 5. Conclusions

In summary, we propose that a combination of metabolomics technologies with natural product discovery processes will be beneficial on multiple levels. Firstly, by increasing the number of identifications in our metabolomics data we may provide novel structures to be tested for bioactivity for any disease under investigation. Multi-parallel analysis using metabolomics technologies will also enhance and increase throughput of chemical characterization processes of many different species from natural resources. Secondly, as mentioned above, natural product chemists have collected a lifetime of compound libraries of active and also inactive pure compounds which can now be analyzed to construct mass spectral and NMR spectral libraries and therefore improve biological interpretations of metabolomics data. The advancements in analytical instrumentation and sophisticated hyphenation of separation techniques with high sensitive detectors have allowed for greater detection of small molecule compounds measurable in biological systems (*i.e.*, primary and secondary metabolites) and undoubtedly will now be used to advance the discovery of natural product chemistry to identify potential novel drugs candidates which will assist in sustaining health and the fight against disease and illness.
